# Supplementary material for: Genome-wide identification of essential genes in the invasive Streptococcus anginosus strain
Source: Sci Rep. 2025 Sep 25;15:32863. doi: 10.1038/s41598-025-18002-0 (PMC12464299; doi:10.1038/s41598-025-18002-0)
Supplement: Supplementary file 2 — Supplementary Material 2 [file 41598_2025_18002_MOESM2_ESM.pdf]

Supplemental File S2

The IS*S1* insertion density plot across the *S. anginosus* 980/01 genome from triplicate transposon library (BHI-A, BHI-B, BHI-C). The bottom line represents the *S. anginosus* 980/01 genome, annotated with coordinate scale.

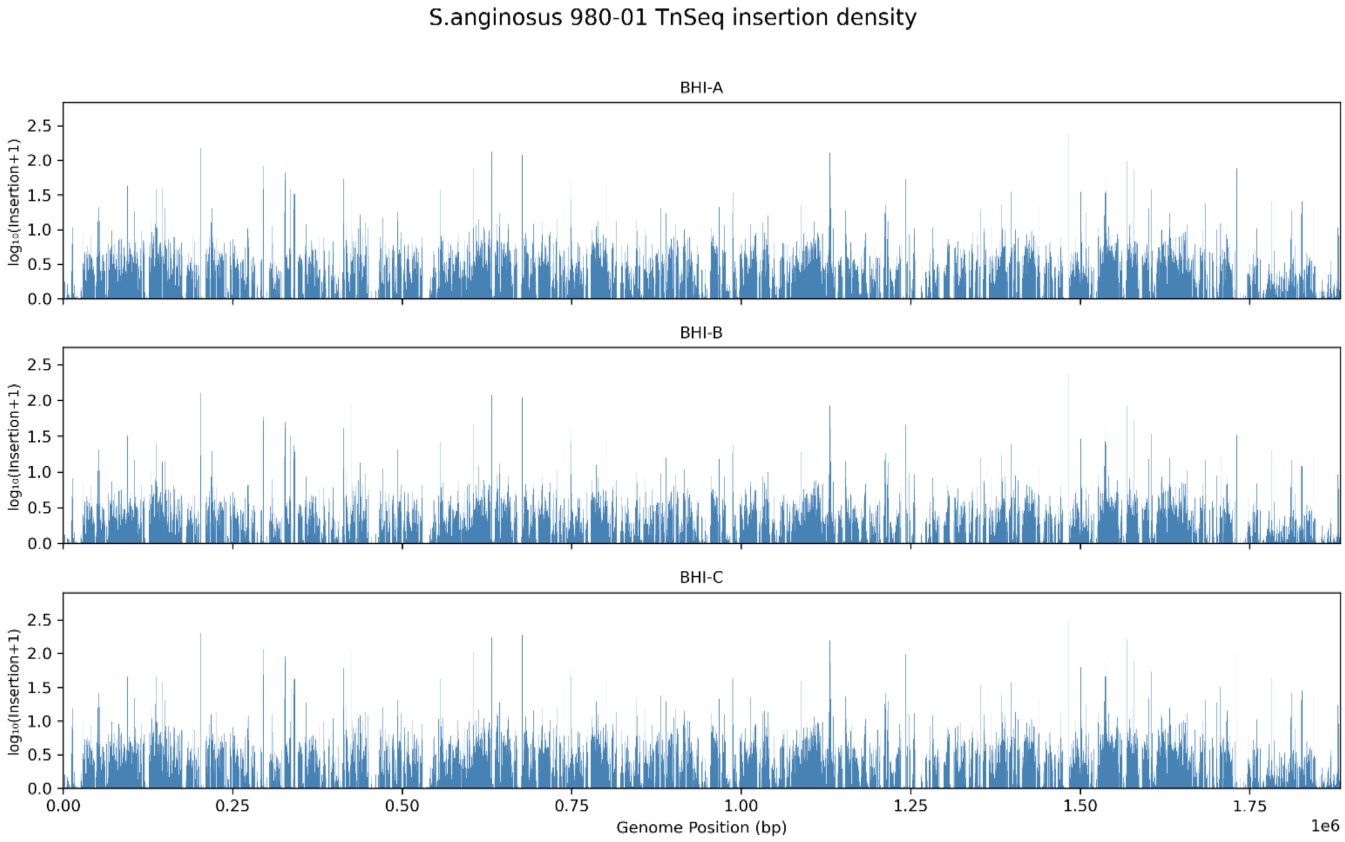

Supplemental File S3

IS*S1* insertion plot for selected *S. anginosus* 980/01 strain-specific essential genes:

a/ *sodA* encoding superoxide dismutase,

b/ *ssaC* encoding the substrate-binding component of a manganese ABC transporter (SsaABC),

c/ *purB* encoding adenylosuccinate lyase,

d/*clpC* encoding ATPase subunits of the Clp protease complex,

e/ *clpX*, encoding ATPase subunits of the Clp protease complex,

f/ *ldh* encoding L-lactate dehydrogenase

Visualization generated using the Integrative Genomics Viewer: the number of reads per IS*S1* insertion is shown for the selected locus. The top lines depict represents a schematic of the *S. anginosus* 980/01 genome, annotated with coordinate scale. The open reading frames are shown as bars, with arrowheads indicating transcriptional orientation.

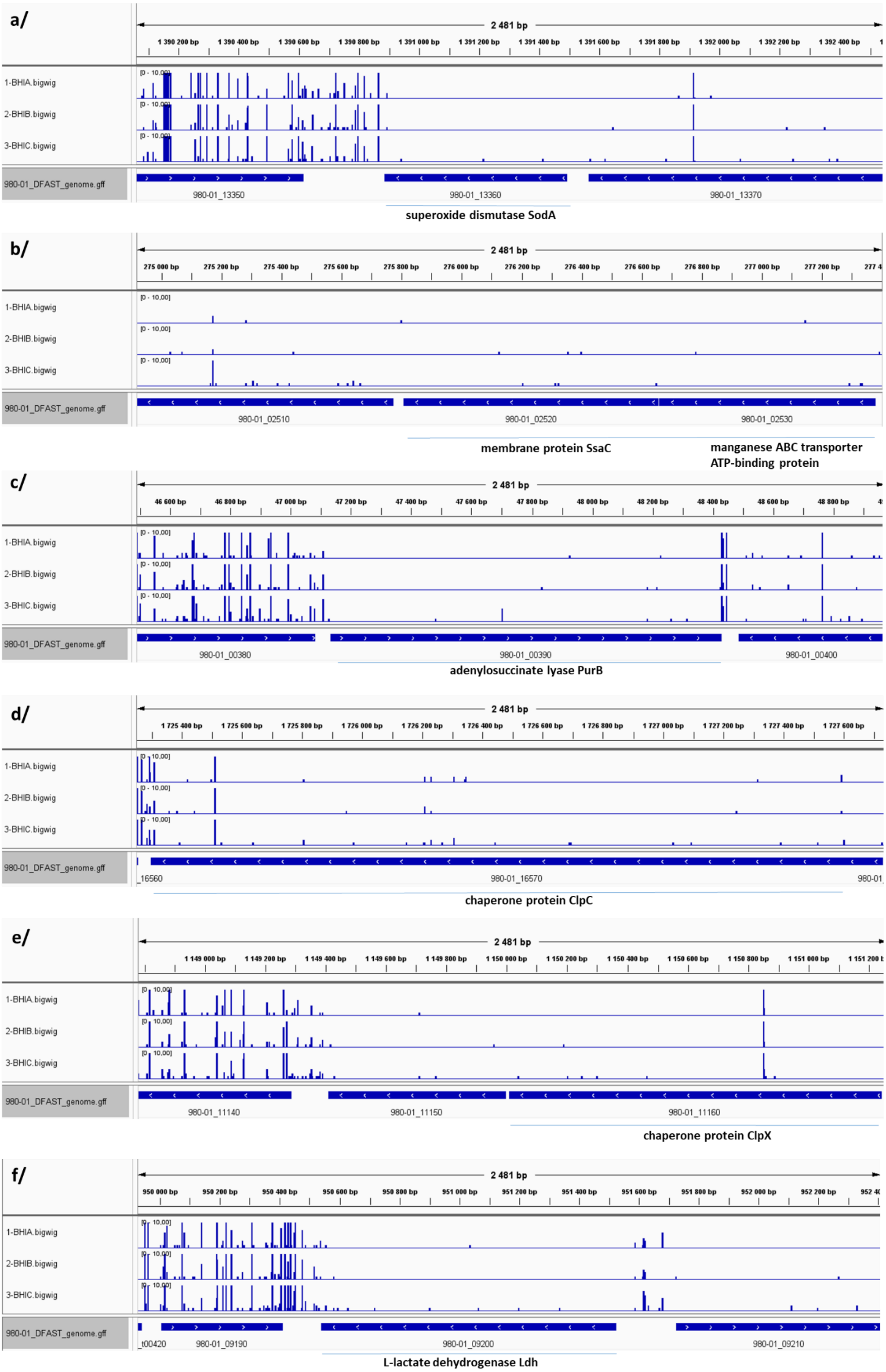

a/
2 481 bp
1 390 200 bp
1 390 400 bp
1 390 600 bp
1 390 800 bp
1 391 000 bp
1 391 200 bp
1 391 400 bp
1 391 600 bp
1 391 800 bp
1 392 000 bp
1 392 200 bp
1 392 400 bp
1-BHIA.bigwig
2-BHIB.bigwig
3-BHIC.bigwig
980-01_DFAST_genome.gff
980-01_13350
980-01_13360
980-01_13370
superoxide dismutase SodA
b/
2 481 bp
275 000 bp
275 200 bp
275 400 bp
275 600 bp
275 800 bp
276 000 bp
276 200 bp
276 400 bp
276 600 bp
276 800 bp
277 000 bp
277 200 bp
1-BHIA.bigwig
2-BHIB.bigwig
3-BHIC.bigwig
980-01_DFAST_genome.gff
980-01_02510
980-01_02520
980-01_02530
membrane protein SsaC
manganese ABC transporter ATP-binding protein
c/
2 481 bp
46 600 bp
46 800 bp
47 000 bp
47 200 bp
47 400 bp
47 600 bp
47 800 bp
48 000 bp
48 200 bp
48 400 bp
48 600 bp
48 800 bp
1-BHIA.bigwig
2-BHIB.bigwig
3-BHIC.bigwig
980-01_DFAST_genome.gff
980-01_00380
980-01_00390
980-01_00400
adenylosuccinate lyase PurB
d/
2 481 bp
1 725 400 bp
1 725 600 bp
1 725 800 bp
1 726 000 bp
1 726 200 bp
1 726 400 bp
1 726 600 bp
1 726 800 bp
1 727 000 bp
1 727 200 bp
1 727 400 bp
1 727 600 bp
1-BHIA.bigwig
2-BHIB.bigwig
3-BHIC.bigwig
980-01_DFAST_genome.gff
_16560
980-01_16570
chaperone protein ClpC
e/
2 481 bp
1 149 000 bp
1 149 200 bp
1 149 400 bp
1 149 600 bp
1 149 800 bp
1 150 000 bp
1 150 200 bp
1 150 400 bp
1 150 600 bp
1 150 800 bp
1 151 000 bp
1-BHIA.bigwig
2-BHIB.bigwig
3-BHIC.bigwig
980-01_DFAST_genome.gff
980-01_11140
980-01_11150
980-01_11160
chaperone protein ClpX
f/
2 481 bp
950 000 bp
950 200 bp
950 400 bp
950 600 bp
950 800 bp
951 000 bp
951 200 bp
951 400 bp
951 600 bp
951 800 bp
952 000 bp
952 200 bp
1-BHIA.bigwig
2-BHIB.bigwig
3-BHIC.bigwig
980-01_DFAST_genome.gff
_t00420
980-01_09190
980-01_09200
980-01_09210
L-lactate dehydrogenase Ldh
